# An Update on Vitamin E, Tocopherol and Tocotrienol—Perspectives

**DOI:** 10.3390/molecules15042103

**Published:** 2010-03-24

**Authors:** Maria Laura Colombo

**Affiliations:** Department of Drug Science and Technology, University of Torino, via Pietro Giuria 9, 10125 Torino, Italy; E-Mail: marialaura.colombo@unito.it.

**Keywords:** vitamin E, tocopherols, tocotrienols, tocochromanols, plant biology, human health

## Abstract

Vitamin E, like tocotrienols and tocopherols, is constituted of compounds essential for animal cells. Vitamin E is exclusively synthesized by photosynthetic eukaryotes and other oxygenic photosynthetic organisms such as cyanobacteria. In order to prevent lipid oxidation, the plants mainly accumulate tocochromanols in oily seeds and fruits or in young tissues undergoing active cell divisions. From a health point of view, at the moment there is a great interest in the natural forms of tocochromanols, because they are considered promising compounds able to maintain a healthy cardiovascular system and satisfactory blood cholesterol levels. Some evidence suggests that the potency of the antioxidant effects may differ between natural or synthetic source of tocochromanols (vitamin E).

## 1. Introduction

Vitamin E is a fat-soluble vitamin, essential for health. It can be stored by the body, so vitamin E does not have to be consumed every day. The vitamin E, *i.e.* chroman-6-ols collectively tocochromanols (tocopherols + tocotrienols), is generally ingested along with fat-containing foods [1]. Good source are vegetable oils, nuts and nut oil seeds, egg yolk, margarine, cheese, soya beans, wheat germ, oatmeal, avocados, olives, green leaf vegetables, *etc.* [2]. Tocopherols are predominant in olive, sunflower, corn, soya beans oils, and tocotrienols are the major vitamin E components of palm oil, of barley and rice bran [3,4].

A very high number of vitamin E publications have appeared over the past 40–50 years. Many literature data are specific for α-tocopherol, while the other forms such as the tocotrienols remain poorly understood. The abundance of α-tocopherol in the human body and the comparable efficiency of all vitamin E molecules as antioxidants led to a neglect the non-tocopherol vitamin E molecules as topics for basic and clinical research. The tocotrienol subfamily of natural vitamin E possesses powerful neuroprotective, anticancer, and cholesterol-lowering properties that are often not exhibited by tocopherols. Current developments in vitamin E research clearly indicate that members of the vitamin E family are not redundant with respect to their biological functions. α-Tocotrienol, γ-tocopherol, and δ-tocotrienol have emerged as vitamin E molecules with functions in health and disease that are clearly distinct from that of α-tocopherol. At nanomolar concentration, α-tocotrienol, not α-tocopherol, prevents neurodegeneration [5].

The review is articulated in paragraphs focusing on the chemistry and the biosynthesis of vitamin E, and the importance of tocochromanols in the human diet. Then a survey on tocopherols and tocotrienols activity on human health is presented. Due to the difficulty in proving the efficacy of vitamin E supplementation and in order to describe evidence-based medicine results, this review provides data mainly obtained from a survey of clinical trials or systematic reviews: The Cochrane Library and clinical trials web site resources.

## 2. Vitamin E Chemistry and Biochemistry

**Figure 1:**
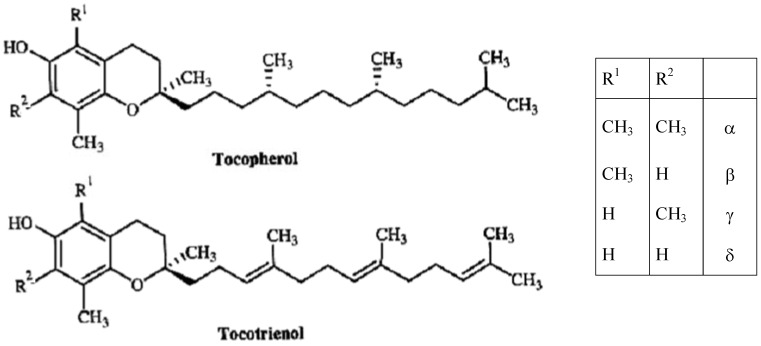
Tocopherol and tocotrienol structures.

Use of the term “vitamin E” term is recommended as the generic descriptor for all tocol and tocotrienol derivatives exhibiting qualitatively the biological activity of alpha-tocopherol [6]. The term “tocopherol” refers to the methyl-substituted derivatives of tocol and is not synonymous with the term vitamin E. Natural tocochromanols comprise two homologous series: the tocopherols with a saturated side chain, and the tocotrienols with an unsaturated side chain (Figure 1). The tocochromanol vitamin E homologues with the largest diffusion in nature are four tocopherols and four tocotrienols: α−, β−, γ− and δ−tocopherol and α−, β−, γ− and δ−tocotrienol [7]. Tocopherols and tocotrienols have the same basic chemical structure characterized by a long chain attached at the 2-position of a chromane ring. Tocotrienols differ from tocopherols because they possess a farnesyl rather than a saturated isoprenoid C_16_ side chain. Either tocopherols or tocotrienols may differ in the methylation of the chroman head group: β− and γ− are structural isomers (5,8-dimethyltocol and 7,8-dimethyltocol), while α− and δ− (5,7,8-trimethyltocol and 8-methyltocol) differ from each other and from β− and γ− because they possess either one more or one less methyl group in the aromatic ring [7]. The only naturally occurring stereoisomer of α-tocopherol hitherto discovered had the configuration 2*R*,4′*R*, 8′*R*; the same in the case for β−, γ− and δ−tocopherol [8].

Tocopherol biosynthesis proceeds only in the photosynthetic organisms, and in particular at the inner envelope membrane of chloroplasts. In this way, photosynthetic apparatus can be protected from oxygen toxicity and lipid peroxidation. The main features of the biosynthetic pathway of tocopherols in plants have been elucidated several years ago using classical biochemical methods. More recently, the tocopherol cyclase enzyme has been identified as a key enzyme of tocopherol biosynthesis. In the last decade much effort has been currently aimed at identifying the genes involved in tocopherol biosynthesis, in order to improve vitamin E levels in crop photosynthetic plants by metabolic engineering [1,9]. Another possibility is to induce vitamin E biosynthesis in non-photosyntetic organisms, usually not able to produce these compounds. Recently, the heterologous synthesis of tocochromanols was performed in *Escherichia coli *strains, a good non-photosynthetic host organism [10,11].

Tocopherol molecules contain three chiral stereocenters at C-2, C-4′ and C-8′ making possible eight stereoisomers (Figure 1). α−Τocopherol occurs in nature is a single stereoisomer *RRR*−α−tocopherol, while synthetic vitamin E is usually a mixture of all eight stereoisomers (all-*racemic*, all-*rac*) in equal proportions and with different biopotency [12]. Based on animal studies comparing natural (*RRR*) and synthetic (all-*rac*) α−tocopherol, a biopotency ratio (natural/synthetic) of 1.36:1 has been derived [13]. Tocotrienols possess only the chiral stereocenter at C-2 (Figure 1) and naturally occurring tocotrienols exclusively possess the (2*R*,3′*E*,7′*E*) configuration [14]. The chirality of these molecules should be taken into consideration when we have to evaluate the activity of a compound in biological studies or clinical trials. Receptors and enzymes in the body are highly stereoselective and only interact with one of the enantiomers of a chiral molecule in a process called chiral recognition. As a result, one enantiomer has the desired effect on the body, while the other may have no effect or an adverse effect [15], and vitamin E vitamers are not interconvertible in the human body [16].

All tocochromanols are amphipathic molecules: the lipophilic isoprenoic side chain is associated to the membrane lipids and the polar chromanol ring is exposed to the membrane surface. The role of tocopherols in photosynthetic organisms has yet to be fully determined. Their antioxidant function is attributed to inhibition of membrane lipid peroxidation and scavenging of reactive oxygen species, but also other functions have been shown in plant metabolism such as role in sugar export from leaves to phloem [10]. In cyanobacteria a protective function against photooxidative damage in photosystem II has been suggested [17]. 

### 2.1. Tocopherol and tocochromanol analysis

In fatty materials the tocochromanol (tocopherol and tocotrienol) analysis, at present time, is carried out preferably by high performance liquid chromatography usually coupled with UV (usually 292 nm) and/or fluorescence detection (usually ex. = 295 nm, em. = 325 nm) at fixed wavelengths or electrochemical detection at a fixed oxidation/reduction potential [18,19,20,21,22]. The fixed parameters of the analysis don’t permit evaluation of the tocopherol and the tocotrienol isomers inside the plant material. In more recent analytical approaches, the analyses were carried out on fatty extracts by means of HPLC coupled with a coulometric array ElectroChemical Detector (ECD). Due to the specific high selectivity of the detector, the crude extract can be directly injected without any preliminary treatment (e.g., saponification) and as a result the tocochromanol isomers can be detected in animal and plant samples [23,24]. 

## 3. Vitamin E Biological Activities

### 3.1. Vitamin E in the diet and the European Community legislation

The role of vitamin E in the human body is not clearly established, but it is known to be an essential compound in some vertebrate species, including humans [25]. Vitamin E intake has been evaluated by the Scientific Committee on Food (SCF) who set a tolerable upper intake level (UL) of vitamin E (as d-α-tocopherol) for adults of 300 mg α-tocopherol/equivalents day. The Joint Expert Committee on Food Additives (JECFA) has defined an acceptable daily intake (ADI) of 0.15–2.0 mg/kg bw/day calculated as α-tocopherol [26]. A recent European Directive on nutrition labelling for foodstuffs as far as the recommended daily allowances (RDA), revised the RDA value for vitamin E: 12 mg value and it defines a rule of what constitutes a “significant amount”. The purpose of this RDA amount is to provide a value for nutrition labelling and the calculation of what constitutes a significant amount [27]. On 30^th^ November 2009 the UE Commission, after consulting the European Food Safety Authority, revised the lists of vitamins and minerals and their forms that can be added to foods, including foods supplements, and a Regulation to this effect has been published. This Regulation shall be binding in its entirety and directly applicable in all Members States [28].

In order to improve and develop the new EPIC Nutrient Database (ENDB) in the European Prospective Investigation into Cancer (EPIC) and Nutrition cohort, a survey among 27 centres in 10 countries was conducted. In both men and women in the European countries, the overall intake of vitamin E showed an interesting difference by European region: higher intake in the South, lower intake in the North, and by Body Mass Index (BMI): higher intake with lower BMI. These observations may be related to the food sources of vitamin E, which is primarily derived from vegetable oils. The gradient of intake by BMI may be similarly related varying dietary patterns of food sources vitamin E. These observations may provide a basis for further studies exploring potential aetiological links between the intake of these nutrients and chronic disease risk in these countries [29].

### 3.2. Antioxidant activity

Tocochromanols are the most effective group of lipophilic phenolic antioxidants. Researchers theorize that antioxidants protect key cell components by neutralizing free radicals before they can cause lipid oxidation or DNA damage. By reducing free radical attack, antioxidants break the chain reaction of lipid peroxidation (*chain-breaking antioxidant*) and they protect the cell membranes by lipid repair and lipid replacement. In this way they may prevent cancer or heart disease. Epidemiological evidences indicate that diet-derived antioxidants, e.g., vitamins A, C, and E, may be important in maintaining human and animal health [30,31].

More recent research demonstrated that tocotrienols play a specific role which goes beyond their known vitamin E antioxidant activity [32,33]. Conflicting results regarding the effects of vitamin E supplementation in reducing levels of free radical damage have been reported from randomized, controlled human trials. This was most likely due to individual response differences. Epidemiologic evidence has indicated that high plasma concentrations of vitamin E are associated with a lower risk of cardiovascular disease and certain types of cancer. In a randomized, double-blinded, placebo-controlled study was determined the effect of a mixture of α- , β- , γ- and δ- tocotrienols and α-tocopherol (total tocotrienol isomer ratio compared with tocopherol is 74%: 26% ca.) on DNA damage, usually considered a target compound in oxidation process. The results obtained suggested that supplementation with this vitamin E mixture reduced the level of DNA damage in healthy subjects. This observation may indicate a possible relation between the molecular mechanisms involved in formation and repair of DNA breaks with tocochromanol mixture supplementation [34].

It has been suggested that a low dietary intake of antioxidant vitamins and minerals increases the incidence rate of cardiovascular disease and cancer. The Supplementation en Vitamines et Mineraux Antioxidants (SU.VI.MAX) study is a randomized, double-blind, placebo-controlled primary prevention trial. A total of 13,017 French adults were included. All participants took a single daily capsule of a combination of 120 mg of ascorbic acid, 30 mg of vitamin E, 6 mg of β-carotene, 100 mug of selenium, and 20 mg of zinc, or a placebo. Median follow-up time was eight years: from 1994 to 2002. The SU.VI.MAX. study tested the efficacy of supplementation with a combination of antioxidant vitamins and minerals, at nutritional doses, in reducing the cancer incidence in a general population not selected for risk factors. Low-dose antioxidant supplementation lowered the total cancer incidence in men only. Finally, the effect of antioxidant supplementation on the incidence of cancer could depend on baseline antioxidant status (which differs from gender and/or nutritional status) and the health status of subjects (healthy *versus* cancer high-risk subjects) [35,36].

### 3.3. Vitamin E and cardiovascular diseases

Supplementary vitamin E was reported to be effective in reducing atherosclerosis progression in subjects with previous coronary artery bypass graft surgery not treated with lipid-lowering drugs. However recent large interventional clinical trials had not shown cardiovascular benefits by vitamin E supplementation. Previous clinical studies used tocopherol as their supplements of vitamin E. In the referred randomized placebo-controlled, blinded end point clinical study a mixture of tocotrienol and α-tocopherol (23.5%) from barley was given to healthy subjects (placebo, 50, 100, 200 mg tocotrienols daily for two months). The aims were determine the bioavailability of single tocohromanol and arterial compliance in healthy subjects. Decreased arterial compliance or increased arterial stiff-ness is a predictor of cardiovascular events not only in diseases but also in normal subjects. The results indicate that single tocotrienols were bioavailable six hours after supplementation from the lower dose and that there was a trend of improved arterial compliance in all treated groups with two months of tocochromanols intake [37]. 

Tocotrienols in animal cells inhibit cholesterol biosynthesis by suppressing 3-hydroxy-3-methyl glutarylCoA reductase enzyme (HMGR): the key-enzyme in the sterologenic pathway, resulting in less cholesterol being manufactured by liver cells. The tocotrienol activity is a post-transcriptionally downregulation of the key-enzyme involved in cholesterol biosynthesis. [38]. 

Lovastatin, a hypocholesterolemic agent useful in the management of hypocholesterolemia, and the tocotrienols have been demonstrated to have cholesterol-lowering properties in animal and humans, but with different mechanisms. The described study, double-blind, cross-over, controlled clinical trial, was carried out in hypercholesterolemic subjects (serum total cholesterol level >5.7 mmol/L) to evaluate the efficacy of therapy: low dose of lovastatin alone (10 mg/day) or plus a minimum effective dose of tocotrienol mixture (50 mg/day). The tocotrienol mixture was obtained from rice bran and it is “generally regarded as safe” (GRAS) [39] and has no known side effects, whereas lovastatin has side effects. The study demonstrated that the low dose of tocotrienol mixture, in combined therapy with lovastatin, was an effective reducing cholesterol agent avoiding some adverse effects of statins [40].

### 3.4. Vitamin E and cancer

Current developments in vitamin E research clearly indicate that members of vitamin E family are not redundant with respect to their biological function. α- , γ- and δ-tocotrienol have emerged as vitamin E molecules with functions clearly distinct from that of α-tocopherol in anticancer activity too. Some data refers that tocotrienols possess antiproliferative and apoptotic activities on normal and cancer human cells [41]. The mechanism could be related to induction of apoptosis mainly via mitochondria-mediated pathway and to cell cycle arrest due to suppression of cyclin D by tocotrienols [42]. Tocotrienols also inhibit vascularization-reducing proliferation, and malignant proliferation demands elevation of HMG-CoA reductase activity and tocotrienols suppress its activity. Only few clinical trials were carried out to determine the effects of tocotrienols on cancer prevention or treatment [43].

In order to test the role of the other half of the natural vitamin E, the tocopherols, on cancer prevention, it was considered the hypothesis that increased intakes of α-tocopherol (AT) and/or β-carotene (BC) prevent lung cancer and possibly other cancers, a randomized, double-blind, placebo-controlled, chemoprevention trial α-tocopherol, β-carotene ATBC Lung Cancer Prevention Study was conducted [44]. The study was performed in southwestern Finland as a joint project between the National Public Health Institute of Finland and the U.S. National Cancer Institute, between 1985 and 1993, on 29,133 eligible male smokers. For AT, the selected dose for the study was 50 mg/day, the dose for BC was 20 mg/day. ATBC researchers reported that men who took β-carotene had an 18% increased incidence of lung cancers and an 8 % increased overall mortality. Vitamin E had no effect on lung cancer incidence or overall mortality. The men taking both supplements had outcomes similar to those taking β-carotene alone [45].

A report published in 2005 finds no clear evidence that men and women who had vascular disease or diabetes and who took 400 I.U. of vitamin E daily for seven years reduced their risk of cancer compared to others with these conditions who took a placebo [46]. The study was not large enough to determine if vitamin E could prevent specific cancers. The report also showed that those taking vitamin E had a 13 percent increased risk of heart failure, a condition in which the heart’s ability to pump blood is weakened. The report comes from a clinical trial called the Heart Outcomes Prevention Evaluation Study Extension (HOPE-TOO). These results emphasize the need to study vitamins and other natural products prior to making public health recommendations. 

Vitamin E supplements, 6.000 I.U./day, do not protect apparently healthy women aged 45 or older against cancer, according to the 10-year randomized Women’s Health Study conducted on 39.876 subjects. It remains unclear whether vitamin E might yet prove protective in other groups of people [47]. 

Tocopherols can be used in order to reduce the potential toxicity due to intake of 13-*cis*-retinoic acid in subjects at high risk for lung cancer. Exposure to tobacco smoke is the major cause of lung cancer. About 50% of new lung cancers arise in former smokers and effective chemoprevention strategies are critical to reduce risk, especially in this large group of subjects. Chemoprevention is an emerging field whereby drug therapy is used to halt or reverse the carcinogenesis process before the emergence of invasive cancer. Based on epidemiological data showing that lung cancer patients have lower serum levels of several antioxidant vitamins, several randomized trials were conducted in order to evaluate beta-carotene, alpha-tocopherol and other vitamins and mineral supplementation. 13-cis-retinoic acid was selected because has reversed the preinvasive lesion, leukoplakia in other clinical trials. At high doses the toxicity is a problem, but at doses ranging from 30 to 70 mg/d it was well-tolerated. A secondary objective of this trial was to determine if α-tocopherol could reduce the toxicity due to 13-*cis*-retinoic acid presence; this hypothesis was based on previous clinical trials conducted on patients with cancer other than lung. The adult subject enrolled in the trial were at high risk for lung cancer with sputum atypia and they received 13-*cis*-retinoic acid at 50 mg/d. p.o.; the same dose of 13-*cis*-retinoic acid was supplemented with α-tocopherol 800 mg/d p.o. or no treatment. The first evidence was the α-tocopherol did not reduce the adverse effects of 13-*cis*-retinoic acid. The 13-*cis*-retinoic acid had a minor but not statistically significant improvement in the histological appearance by the dysplasia index [48].

## 4. Conclusions

Vitamin E is an interesting group of compounds, able to exert many and different biological activities in plant, animal and human cells, but the physiological and/or pharmacological role in cell life it is not yet fully described. There are many literature data having positive or negative results on the same biological activity. Vitamin E deficiency is rare in humans, although it may develop in premature infants and in persons with a chronic malabsorption of fats, as well as mild anemia, ataxia and pigmentary changes in the retina. We can observe that vitamin E compounds have to be better evaluated for their properties, as underlined in a commentary on updating information about vitamin E [49].
